# Treatment of xerostomia in Sjögren’s syndrome – what effect does it have on the oral microbiome?

**DOI:** 10.3389/fcimb.2025.1484951

**Published:** 2025-04-14

**Authors:** Damian Muszyński, Robert Kucharski, Natalia Marek-Trzonkowska, Magdalena Kalinowska, Aleksandra Brzóska, Marika Bolcewicz, Leszek Kalinowski, Karolina Kaźmierczak-Siedlecka

**Affiliations:** ^1^ Scientific Circle of Studies Regarding Personalized Medicine Associated with Department of Medical Laboratory Diagnostics, Medical University of Gdansk, Gdansk, Poland; ^2^ Department of Medical Laboratory Diagnostics – Fahrenheit Biobank BBMRI.pl, Medical University of Gdansk, Gdansk, Poland; ^3^ Neodentica Dentistry Center, Gdansk, Poland; ^4^ International Centre for Cancer Vaccine Science, University of Gdansk, Gdansk, Poland; ^5^ Laboratory of Immunoregulation and Cellular Therapies, Department of Family Medicine, Medical University of Gdansk, Gdansk, Poland; ^6^ University of Social Sciences and Humanities, Warsaw, Poland; ^7^ BioTechMed Center, Department of Mechanics of Materials and Structures, Gdansk University of Technology, Gdansk, Poland

**Keywords:** Sjögren’s syndrome, oral microbiome, xerostomia, pilocarpine, cevimeline, artificial saliva

## Abstract

Sjögren’s syndrome is an autoimmune disease characterized by lymphatic infiltration of secretory tissues. The disease results in dryness of the eyeball or mouth, which often occur simultaneously. Agents used to treat Sjögren’s syndrome may improve oral hydration and the patient’s quality of life. There are several pharmacological and non-pharmacological agents used to treat significant problem like xerostomia. The use of appropriate medicines (i.e. pilocarpine and cevimeline) may cause changes in the local microbiome, which is very sensitive to quantitative changes in water. As a result of Sjögren’s syndrome, a new balance of the microbiome is established in the oral cavity, which, if disturbed by medical measures, may increase the risk of oral lesions (such as periodontopathies or caries) or reduce this risk. Overall, the knowledge regarding microbiological aspects and agents treating oral dryness is still not well described but initial results indicate some microbial alterations.

## Introduction

Sjögren’s syndrome (SS) is a systemic chronic autoimmune disease, which touches connective tissues (André and Böckle, 2022). It has been established that 1-3% of the general population may suffer from this disease (Bayetto and Logan, 2010). Often, it is diagnosed between the 5th and the 7th decade of life and occurs eight times more frequently (about eight times more often) in women than in men (Bayetto and Logan, 2010; André and Böckle, 2022). The etiology of this disease is unknown, but environmental factors are suspected to play a key role (André and Böckle, 2022). Risk factors include viral diseases (Epstein-Barr virus, HCV, human lymphocyte leukemia virus), genetic predisposition and sex hormones (Bayetto and Logan, 2010). However, the greatest impact appears to be exerted by the Epstein-Barr virus, which is found in biopsy materials taken from the lacrimal glands, as well as in salivary gland and saliva specimens (Negrini et al., 2022).Viral infection most likely promote the production of auto-antibodies, ultimately leading to cross-reactivity of immune elements with host antigens (Bayetto and Logan, 2010). It is assumed that the genetic predisposition to Sjögren’s syndrome can be diagnosed if this disease occurs in at least two family members (Bayetto and Logan, 2010). The relationships between polymorphic major histocompatibility complex (MHC) genes are expected to play an important role here (Bayetto and Logan, 2010).The disproportion in the incidence of SS allows us to put forward the theory that sex hormones play an important role in etiology. Research show that estrogens influence the immune response by increasing the production of antibodies by B cells. The menopause period in women is the most common age range in which SS occurs, which may indicate an indirect effect of estrogens on the development of this disease (Bayetto and Logan, 2010).

Sjögren’s syndrome could be primary (pSS) or secondary (sSS) but both is likely similar (Bayetto and Logan, 2010). Their difference resides in that sSS is an element of other autoimmune diseases, most often systemic lupus erythematosus (15-36%), rheumatoid arthritis (20-32%) or progressive and limited systemic sclerosis (11-24%) (Stefanski et al., 2017). pSS is not correlated with any autoimmune diseases (Bayetto and Logan, 2010). There are general symptoms of dryness, known as dryness syndrome (xerostomia, xerophthalmia) (Bayetto and Logan, 2010; Zeron et al., 2013). The pathogenesis is still unknown (Negrini et al., 2022). However, the main role in the development might be played by elements of the immune system (T and B cells) (Bayetto and Logan, 2010; Tian et al., 2021). Excessive activation of B cells located in the exocrine glands causes the production of anti-SSA and anti-SSB autoantibodies (0–70% patients with pSS) (Bayetto and Logan, 2010; Tian et al., 2021). T cells, on the other hand, infiltrate the exocrine glands and increase the extremity of cytokines, thus leading to damage to the lobular cells and ultimately to xerostomia (Tian et al., 2021).

The concerns of Sjögren’s syndrome center on disorders of the exocrine glands (Tian et al., 2021). Most commonly, patients report symptoms such as dry eyes and dry mouth (89% of them experience both) (Negrini et al., 2022). In the aspect of the oral cavity, dysphagia, pain and burning are frequently noticeable, which is related to reduced saliva production (Negrini et al., 2022). Often, a physical examination also reveals dental caries and periodontal disease, as well as frequently recurrent *Candida albicans* infections (occurring 10 times more often than in the normal population) (Stefanski et al., 2017; Negrini et al., 2022). In sSS, symptoms of fatigue and joint pain may also manifest (6). Usually treating Sjögren’s syndrome is symptomatic (Baer and Walitt, 2018). It is crucial to stay hydrated, quit smoking, apply fluoride to prevent dental caries and avoid fatigue (among others, good sleep hygiene) (Baer and Walitt, 2018). Pharmacological treatment typically relies on muscarinic agonists, such as pilocarpine and cevimeline, which enhance the production of saliva and tears (Baer and Walitt, 2018). However, these drugs have cholinergic side effects, such as flushing chills, and excessive sweating (Baer and Walitt, 2018). To prevent this, moisturization of the oral tissues can also be achieved by using artificial saliva (Bayetto and Logan, 2010).

## Oral healthy microbiome

The oral microbiome, being one of the most diverse microbial communities in the human body, plays a crucial role in maintaining human health (Zhou et al., 2023). The human oral microbiome encompasses all microorganisms found in the oral cavity—including distinct habitats such as teeth, gingival sulcus, attached gingiva, tongue, cheek, lip, hard palate, and soft palate—and its contiguous extensions like the tonsils, pharynx, esophagus, Eustachian tube, middle ear, trachea, lungs, nasal passages, and sinuses, though most studies and samples focus on the oral cavity itself (Dewhirst et al., 2010). The oral cavity contains over 700 different species of bacteria, fungi, viruses, archaea, and protozoa. Bacteria are the most extensively studied microorganisms in the oral cavity, yet only 57% of the bacterial species present have been named (Kozak and Pawlik, 2023). In healthy individuals, the oral microbiome predominantly comprises facultative anaerobic Gram-positive bacteria (Kozak and Pawlik, 2023). 16S rDNA profiling of a healthy oral cavity identified six major bacterial phyla: *Firmicutes, Actinobacteria, Proteobacteria, Fusobacteria, Bacteroidetes*, and *Spirochaetes* (Verma et al., 2018). Brief descriptions of the more important bacteria are provided below.

One of the most important representatives of *Firmicutes* is *Streptococcus mutans*, which lives mainly on tooth surfaces (Lemos et al., 2019; Sedghi et al., 2021). Its ability to metabolize carbohydrates, create low-pH acids and finally produce glucans (an important component for the backbone of dental plaque), which makes it one of the existing etiological factors of caries (Lemos et al., 2019). *Actinomyces*, belonging to *Actinobacteria*, are the natural flora of the oral cavity, living on the surfaces of mucous membranes (Könönen and Wade, 2015). They constitute (*A. oris*, *A. naeslundi*) quite an important element of the oral biofilm, and may also participate in the development of dental caries (Könönen and Wade, 2015). As a result of the microbiological balance in the oral cavity, there may be a sudden increase in the occurrence of *Actinomyces*, causing actinomycosis (Smego and Foglia, 1998; Könönen and Wade, 2015). *Haemophilus*, which belong to the *Proteobacteria*, cause a wide range of infections that require blood-derived factors for their growth (Musher, 1996). A natural component of the oral cavity is *H. aphrophilus*, which under specific conditions can cause bacterial endocarditis (Musher, 1996). *Porphyromonas gingivalis* is an anaerobic gram-negative bacterium, classified as *Bacteroidetes*, strongly associated with periodontal disease (Zhou and Luo, 2019). By regulating the human’s immune response, it disrupts its chomesotasis, which contributes to the occurrence of gum diseases (Zhou and Luo, 2019). These actions are due to a series of protein adhesins, binding proteinases and hemin. More and more often, you can also find information about the connection between *P. gingivalis* and the occurrence of cancers, among others. squamous cell carcinoma of the oral cavity or esophagus (Zhou and Luo, 2019). *Fusobacterium nucleatum* is a Gram-negative rod-shaped bacterium, belonging to *Fusobacteria*, commonly found in the oral cavity. It plays a crucial role in dental plaque biofilm formation (McIlvanna et al., 2021). Although traditionally not considered pathogenic in the oral cavity, it has emerged as an important player in driving inflammation and may act as an opportunistic pathogen in extra-oral sites (McIlvanna et al., 2021). *F. nucleatum* acts as a “bridging” bacterium, facilitating interactions between early and late colonizers in dental plaque and has the potential to bind to various cell types, thus contribute to carcinogenesis through its adhesins FadA and Fap2 (McIlvanna et al., 2021). *Treponema denticola*, belonging to *Spirochaetes* play a significant role in oral diseases such as periodontitis, necrotizing ulcerative gingivitis, and acute pericoronitis (Yousefi et al., 2020). These bacteria contribute to tissue destruction through direct bacterial action and by triggering an exaggerated host inflammatory response (Yousefi et al., 2020).

Bacteria are the primary focus of research on the human oral microbiome and constitute the majority of biomass in the oral environment, whereas fungi are estimated to make up as little as less than 0.1% of it (Baker et al., 2017). The initial study examining the composition of the fungal component oral in healthy individuals’ microbiota found that the most commonly identified fungal genera were *Candida*, *Cladosporium, Aureobasidium, Saccharomycetales*, and *Aspergillus* (Ghannoum et al., 2010). Fungi in the oral cavity have been studied mainly in relation to diseases, and their role in maintaining a healthy oral ecology remains largely unknown (Krom et al., 2014). The review exploring current knowledge on fungi-bacteria interactions likewise highlights the needs for further research to understand fungi’s contribution to oral health (Krom et al., 2014). The oral virome in healthy individuals consists mainly of bacteriophages, which are more numerous than eukaryotic viruses (Baker et al., 2017). Compared to the oral bacteriome, oral virome is characterised by significant individual variability while remaining stable over time (Baker et al., 2017). Despite of technological advancements, studying the human virome is challenging due to the low abundance of viral nucleic acids, the difficulty in purifying unknown virions, and the complexities in analyzing and identifying novel viruses (Bikel et al., 2015). The viruses causing symptoms are well-studied, while the vast, symptomless majority remains poorly understood (Lecuit and Eloit, 2013).

## Oral microbiome in xerostomia of Sjögren’s syndrome

Reduced amount of saliva plays an important role in Sjögren’s syndrome (Bayetto and Logan, 2010; van der Meulen et al., 2018b; Negrini et al., 2022). First, it cleanses the oral cavity - dilutes food remains and washes out bacteria from the surface of the mucous membranes (Lynge Pedersen and Belstrøm, 2019). In SS, the persistence of food and microorganisms is prolonged (sugar clearance is also reduced), which leads to changes in the composition of the flora, promoting the growth of acid-forming and tolerant bacteria. Acidic environment, in particular *Streptococcus mutans, Streptococcus sobrinus* and *Lactobacilli*, which produce acids from retained carbohydrates and increase the risk of developing caries (Li et al., 2022). *S. mutans* may have a close correlation with the occurrence of *Candida albicans* (Li et al., 2022). Moreover, in a study conducted by Nadig et al., which concerned determining the relationship between salivary flow rate and the number of *Candida* in xerostomia, a correlation was observed between low SFR and an increase in the number of *Candida*, in which *C. albicans* was the most common (Nadig et al., 2017). Analysis of the interactions between these two species showed that protein genes related to carbohydrate metabolism in *C. albicans* were enhanced (Li et al., 2022). What’s more, as a result of this culture, the mass of the total biofilm increased (Li et al., 2022). Another issue are mucins present in saliva (Lynge Pedersen and Belstrøm, 2019). They prevent the adhesion and formation of a biofilm by microorganisms such as staphylococci, streptococci or *Candida* and participate in the binding of, for example, *Porphyromonas gingivalis* (Li et al., 2022). The coating formed from saliva is important in preventing the colonization of various surfaces of the oral cavity (Li et al., 2022). The topic of the role of saliva is very complex. We know that its impact on the microbiome is influenced by among others, salivary amylase, the salivary peroxidation system, salivary lysosomes, lactoferrin, rich proteins in proline, staterin, cystatin, histatin and immunoglobulins (Lynge Pedersen and Belstrøm, 2019). In recent years, many studies have been carried out aiming to describe the differences between the microbiome in people with Sjögren’s syndrome, people with xerostomia for other reasons and healthy patients. In a study by Huang YF et al. statistically significant differences were observed - a decrease in the *Bacteroides* genus and a significant increase in the *Firmicutes* genus (Huang et al., 2019). Another study suggests that a high ratio of *Firmicutes* to *Proteobacteria* (taco) may be a characteristic indicator of xerostomia (van der Meulen et al., 2018a). In addition, a higher total number of microorganisms of the genus *Candida*, *Streptococcus mutans*, lactobacilli was noticed (Myers, 2015). Although impaired salivary secretion has been shown to be the dominant contributor to the change in oral microflora in SS, differences were reported in the microbiota between people with xerostomia and Sjögren’s syndrome (van der Meulen et al., 2018b). Similary, the same that could be used in treatment and diagnosis (Sembler-Møller et al., 2019), however not all studies confirm this. Example of which is a lower relative number of *Granulicatella* and *Bergeyella* in patients with compared to Sjögren’s syndrome (van der Meulen et al., 2018a). A study conducted on a small group using PCR methods allowing the division of microorganisms even into their species showed significant differences (Rusthen et al., 2019). First of all, a lower percentage of *Streptococci, Neisseria, Actinomyces lingnae* and *Haemophilus* was observed in people with Sjögren’s syndrome, and a higher percentage of *Porphyromonas pasteri*, *Megasphaera micronuciformis* (Rusthen et al., 2019). The study’s authors emphasize that their findings may in the future be used to detect SS based on the examination of the oral microbiome as a specific biomarker of the disease, and prove that the changes in oral flora in Sjögren’s syndrome are caused not only by reduced salivary secretion, but also by other factors that have not yet been described (Rusthen et al., 2019). Another publication describes *Lactobacillus* and *Streptococcus* as potential biomarkers (Kim et al., 2022). It also suggests that in the future it may be possible to treat the symptoms of Sjögren’s syndrome by using prebiotics, which is already being conducted in the treatment of other autoimmune diseases such as rheumatism (Zamani et al., 2016). Interestingly, there are publications suggesting that changes in the oral microbiome may indirectly lead to the development of Sjögren’s syndrome (Tsigalou et al., 2018). Therefore, monitoring the oral microflora could be more specific in prevention, but the exact pathomechanism of this disease has not yet been clarified (Tsigalou et al., 2018). This is an interesting direction for future research on the links between Sjögren’s syndrome and the oral microbiome (Tsigalou et al., 2018). A comparison of the microbiome in xerostomia with the healthy oral cavity is presented in the **Table 1**.

**Table 1:** Comparison of the occurrence of more important microorganisms in a healthy oral cavity and in xerostomia, taking into account the most important functions of microorganisms.

Microorganism	Healthy oral cavity	Oral cavity in xerostomia (Sjögren’s syndrome)	Function/potential impact
***Streptococcus mutans***	Present but controlled by microbial balance (Sedghi et al., 2021)	Significant increase in numbers (Li et al., 2022)	Metabolizes carbohydrates, produces acids that lower pH, contributes to dental caries, associated with subacute bacterial endocarditis (Lemos et al., 2019)
***Lactobacillus***	Occurs in small quantities (Sedghi et al., 2021)	Increase in numbers (Li et al., 2022)	Produces acids and increases the risk of caries (Struzycka, 2014; Sedghi et al., 2021; Li et al., 2022)
***Actinomyces (Actinomyces oris, Actinomyces naeslundii)***	Natural component of biofilm (Könönen and Wade, 2015)	Decline of *Actinomyces lingnae* (Rusthen et al., 2019)	Co-creates biofilm, may participate in the development of caries, and under specific conditions it may cause actinomycosis (Könönen and Wade, 2015)
***Haemophilus (Haemophilus aphrophilus)***	Present as a component of flora (Musher, 1996)	Decreased abundance (total *Proteobacteria*) (Rusthen et al., 2019)	May cause infections, e.g. endocarditis (Musher, 1996)
***Porphyromonas gingivalis***	May be present in small amounts (Zhou and Luo, 2019; Sedghi et al., 2021)	Increase in numbers (Li et al., 2022)	Associated with periodontal disease, it affects the immune response and possibly some cancers (Zhou and Luo, 2019)
***Fusobacterium nucleatum***	Present, supports biofilm formation (Sedghi et al., 2021)	Increase in numbers (McIlvanna et al., 2021; Li et al., 2022)	Actes as a bridging bacterium in the biofilm and may influence inflammation and tumor formation (McIlvanna et al., 2021)
***Treponema denticola***	Occurs in controlled amounts (Sedghi et al., 2021)	Increase in numbers (total *Firmicutes*) (van der Meulen et al., 2018b; Huang et al., 2019)	Involved in periodontal diseases and destroys tissues (Yousefi et al., 2020)
***Candida albicans***	Occurs in trace amounts (Sedghi et al., 2021)	Possible increase in numbers, especially with low salivation (Nadig et al., 2017; Li et al., 2022)	Associated with fungal infections, it increases the risk of candidiasis (Krom et al., 2014)

## Agents used to treat xerostomia of Sjögren’s syndrome and their impact on the oral microbiome

Sjögren’s syndrome often causes decreased salivation – xerostomia (Bayetto and Logan, 2010; Baer and Walitt, 2018). It is estimated that 88% of patients with SS have decreased salivary secretion, which ultimately resulted in xerostomia in around 75-92% of patients (Cartee et al., 2015). These symptoms can be treated with agents that lead to produce more saliva (sugar-free chewing gum, pilocarpine, cevimyelin) or with artificial saliva, which may play a similar role (Bayetto and Logan, 2010). These pharmacological and non-pharmacological agents are described below.

### Pilocarpine

One of the basic pharmacological agents used to treat xerostomia occurring in Sjögren’s syndrome is pilocarpine (Watanabe et al., 2018). It is a muscarinic agonist that imitates the action of acetylcholine (Bayetto and Logan, 2010). Pilocarpine binds to the muscarinic acetylcholine receptor located in the acinar cells of the salivary glands (Kapourani et al., 2022). It is able to activate all five subtypes of the aforementioned receptors, although the therapeutic effect is mostly related to receptor M3R (Kapourani et al., 2022). After oral administration, there is an increase in the rate of saliva secretion, which may persist for 1-2 hours (Wiseman and Faulds, 1995). Despite saliva stimulation, pilocarpine does not reduce the risk of caries in patients with xerostomia (Hsu et al., 2019). An observational study by Hsu CY et al. among patients suffering from new-onset primary Sjögren’s syndrome, taking pilocarpine and individuals not taking it, showed that the risk of caries does not differ significantly between both studied groups (Hsu et al., 2019). A similar situation could be observed in the case of the risk of periodontitis and oral candidiasis (Hsu et al., 2019). *Candida albicans* is mainly responsible for the occurrence of candidiasis in the oral cavity, and *P. gingivalis* is responsible for periodontitis (Hellstein and Marek, 2019; Di Stefano et al., 2022). Dental caries is caused by bacteria such as *Streptococcus mutans, Actinomyces*, and *Lactobacillus* (Struzycka, 2014). The above-mentioned study shows that the risk of candidiasis, periodontitis and caries does not change significantly, which leads to the conclusion that the use of pilocarpine does not significantly affect the above-mentioned elements of the oral microbiome of patients with SS (Hsu et al., 2019).

### Cevimeline

Another drug used to treat xerostomia occurring in Sjögren’s syndrome is cevimeline (Cevimeline). It is an acetylcholine analogue that stimulates the salivary glands via muscarinic receptors (Bayetto and Logan, 2010; Cevimeline). Cevimeline, similarly as pilocarpine, binds the M3R and M1R receptors (also found in the salivary glands); Their activation intensifies the work of the secretory glands, resulting in an increase in the amount of produced saliva and, consequently, a reduction in the symptoms of xerostomia (Fife et al., 2002). Rose F et al. showed that cevimeline reduces the feeling of subjective dry mouth while increasing saliva flow (Fife et al., 2002). However, there was no data regarding the composition of stimulated saliva or changes in the potential risk of caries or candidiasis in patients with SS (Fife et al., 2002). The effect of cevimeline on the oral microbiome is still unclear. It can only be assumed that, since its effects are similar to those of pilocarpine, cevimeline may also slightly change the oral microbiome in patients with Sjögren’s syndrome. The main difference between cevimyelin and pilocarpine is that the former binds to M2R receptors to a lesser extent, resulting in reduced of cardiac tissue (Bayetto and Logan, 2010). A study conducted by Ono K. et al. comparing the effects of cevimeline and pilocarpine in rats showed that cevimeline and pilocarpine increase Ca(2+) concentration to similar quantities, but the cevimeline increases it at a steeper rate (Ono et al., 2012). What is more, cevimeline inhibits water intake, which is supposed to contrast with the effect of pilocarpine (Ono et al., 2012). Possibly due to its slightly different effect on stimulated saliva, cevimeline may cause changes in the oral microbiome in people with xerostomia.

### Sugar-free gum

Saliva stimulation is also provided by consuming sugar-free chewing gum, which results in dislodging debrits and the mechanical bacterial clearance (Bayetto and Logan, 2010; Maintaining the Oral Health of Patients With Sjögren’s Syndrome, 2018). When chewing it, the gleeking phenomenon increases, during which the muscles of the tongue and the muscles connected to it are used to stimulate saliva, especially in the submandibular and sublingual glands (Maintaining the Oral Health of Patients With Sjögren’s Syndrome, 2018). Moreover, chewing gum neutralize acids due to its baking soda content (Maintaining the Oral Health of Patients With Sjögren’s Syndrome, 2018). Sugar-free chewing gum owes its action to polyalcohols, the most common of which are xylitol, sorbitol, and mannitol (Imfeld, 1994). The most popular ingredient is xylitol – a pentol that is not metabolized by cariogenic bacteria, including *S. mutans*. Critically, xylitol may reduce the presence of these bacteria in the oral cavity and thanks to this, reduce the risk of caries (Imfeld, 1994; Janket et al., 2019). A study conducted by Chen SY et al. regarding xylitol also showed that it has an inhibitory effect on *Porphyromonas gingivalis* - the bacterium responsible for the occurrence of periodontal disease (Chen et al., 2023). However, the inhibitory process itself remains unclear; some sources claim that there is a relationship between the dose of xylitol and the quantitative change of *P. gingivalis*, others have shown an effect on the expression of cytokines induced by *P. gingivalis* (Chen et al., 2023). Instead of xylitol, sugar-free chewing gum can include sorbitol, which is a heksykol often used as a sweetener (Imfeld, 1994; Liauw and Saibil, 2019). Oza S. et al. showed that sorbitol gum is effective in reducing lactic acid bacteria comparably to that of xylitol gum, although it is not as effective in reducing the number of streptococci (Oza et al., 2018). Sorbitol has a reducing effect on *Lactobacillus*, but not comparable to that of xylitol on *S. mutans* (Oza et al., 2018). Regarding mannitol, another hexitol, there is the least amount of research (Imfeld, 1994). JH. Shaw determined that mannitol, like sorbitol, is unable to increase caries activity in rats in the presence of starch. It is also known that mannitol, like other polyalcohols, does not have an acid-forming effect (Imfeld, 1994). All polyalcohols work in a similar way to xylitol, so it is possible that all of them, including mannitolol, reduce the amount of *S. mutans* in dental plaque to some extent (Imfeld, 1994; Janket et al., 2019). However, further research is required to determine the specific effect on the remaining bacteria that can cause caries.

### Artificial saliva

Artificial saliva’s ingredients should resemble the saliva secreted by human salivary glands by adding appropriate remineralizing and antimicrobial agents (Mystkowska et al., 2018; Maintaining the Oral Health of Patients With Sjögren’s Syndrome, 2018). Its closeness to natural saliva depends on the ingredients used to produce it (Niemirowicz-Laskowska et al., 2020). For example, according to the study of Mystkowska J. et al., preparations based mainly on mucin render parameters most similar to those of human saliva (Mystkowska et al., 2018). Niemirowicz-Laskowska K. et al. allows us to see that different preparations inhibit the multiplication of oral pathogens to varying degrees (Niemirowicz-Laskowska et al., 2020). Selected pathogens (fungal or bacterial) incubated on the medium in the presence of tested artificial saliva preparations (Niemirowicz-Laskowska et al., 2020). Interestingly, in the case of only one of the preparations used, a significant reduction in the growth of microorganisms could be observed (Niemirowicz-Laskowska et al., 2020). The preparations had a particular effect on *Pseudomonas auroginosa* and *C. albicans*. However, the impact on *S. mutans* was negligible, as none of the tested preparations influenced its multiplication (Niemirowicz-Laskowska et al., 2020). In the aforementioned study, it was also possible to observe that the use of nanoparticles in artificial saliva preparations reduces the adhesion process by up to 65% for gram-positive bacteria and fungi and by 45% for gram-negative bacteria, which ultimately resulted in a reduction of *E. coli* by 70% and by 40% *P. auroginosa* and *C. albicans* (Niemirowicz-Laskowska et al., 2020). Moreover, the study conducted by Silva MP et al. showed that artificial saliva containing carboxymethylcellulose reduces the presence of *Candida albicans* more effectively than artificial saliva consisting of lactoferrinins, lysozyme, cactoperoxidase (Silva et al., 2012). Nevertheless, artificial saliva still does not provide satisfactory results (Kho, 2014). A ramdomized study conducted by Cifuentes M. et al. showed that the use of pilocarpine is more effective than the use of artificial saliva in people suffering from Sjögren’s syndrome (Cifuentes et al., 2018). A summary of the above information is provided in **Table 2**.

**Table 2:** Comparison of test results for individual anti-xerostomia agents against microorganisms.

Agents	References	Study model	Intervention	Effect of intervention
pilocarpine	Hsu et al. (2019)	people	Two study groups: patients with primary SS taking pilocarpine and patients not taking it	no significant difference between groups in terms of the risk of oral candidiasis, caries, periodontitis
cevimeline	Ono et al. (2012)	rats	Two study groups - the first one was given cewimeline and the second one was given pilocarpine	both agents appear to increase blood flow in the parotid gland, but cevimelin at higher concentrations causes an increase in Ca2+ concentration
sugar-free gum (xylitol)	Chen et al. (2023)	article review	*in vitro* studies will be under review	xylitol has an inhibitory effect on *P. gingivalis*
artificial saliva	Silva et al. (2012)	people	Three study groups - the first was based on artificial saliva with cabboxymethylcellulose, the second on artificial saliva containing glucose oxidase, lactoferrin, lysozyme and lactoperoxidase and the last group used sterile distilled water	in group I, a significant reduction of *C. albicans* was observed compared to group II

### Biological treatment

Biological treatment is an increasingly common method of treating various types of diseases. This also applies to Sjögren’s syndrome, but only under specific conditions (Marinho et al., 2023). For example, rituxinab (RTX) is not recommended for symptoms of dryness, but is recommended as a first-line drug in patients with SS combined with severe general symptoms and a risk of lymphoma (Marinho et al., 2023). It is also possible to recommend the use of RTX as a second-line therapy in cases where the drug has not been previously used and the disease has a severe general course (Marinho et al., 2023). Due to such restrictive use of biological drugs, it is difficult to determine the impact of RTX on the oral microbiome.

## Conclusions

Agents used in the treatment of Sjögren’s syndrome, especially in case of xerostomia, have a real impact on the composition of the oral microbiome. However, some of them, such as pilocarpine, despite providing relief from dry mouth, do not significantly affect the risk of periodontopathy or tooth decay, even though they are considered one of the best means of treating this disease. It should be emphasized that this field is not well discovered. Further research is needed to determine the impact of individual agents, in particular cevimeline, on the oral microbiome. Research on artificial saliva is also necessary due to the general treatment of its effect on xerostomia, without taking into account the division into xerostomia caused by Sjögren’s syndrome, radiotherapy or chemotherapy. This is especially important in the context of the current discussion of similarities and differences in the oral microbiome resulting from the above-mentioned factors.
